# DNA N6-Methyladenine Modification in Wild and Cultivated Soybeans Reveals Different Patterns in Nucleus and Cytoplasm

**DOI:** 10.3389/fgene.2020.00736

**Published:** 2020-07-27

**Authors:** De-Hui Yuan, Jian-Feng Xing, Mei-Wei Luan, Kai-Kai Ji, Jun Guo, Shang-Qian Xie, Yuan-Ming Zhang

**Affiliations:** ^1^Crop Information Center, College of Plant Science and Technology, Huazhong Agricultural University, Wuhan, China; ^2^Key Laboratory of Genetics and Germplasm Innovation of Tropical Special Forest Trees and Ornamental Plants (Ministry of Education), Hainan Key Laboratory for Biology of Tropical Ornamental Plant Germplasm, College of Forestry, Hainan University, Haikou, China

**Keywords:** methylation, nucleus, cytoplasm, wild soybean, cultivated soybean

## Abstract

DNA 6mA modification, an important newly discovered epigenetic mark, plays a crucial role in organisms and has been attracting more and more attention in recent years. The soybean is economically the most important bean in the world, providing vegetable protein for millions of people. However, the distribution pattern and function of 6mA in soybean are still unknown. In this study, we decoded 6mA modification to single-nucleotide resolution in wild and cultivated soybeans, and compared the 6mA differences between cytoplasmic and nuclear genomes and between wild and cultivated soybeans. The motif of 6mA in the nuclear genome was conserved across the two kinds of soybeans, and ANHGA was the most dominant motif in wild and cultivated soybeans. Genes with 6mA modification in the nucleus had higher expression than those without modification. Interestingly, 6mA distribution patterns in cytoplasm for each soybean were significantly different from those in nucleus, which was reported for the first time in soybean. Our research provides a new insight in the deep analysis of cytoplasmic genomic DNA modification in plants.

## Introduction

DNA methylation is one important epigenetic modification that occupies crucial roles in the regulation of gene expression (Lang et al., 2017), embryonic development (Smith and Meissner, 2013), and transposon silencing (Zhang H. et al., 2018). Various DNA methylation types have been reported in eukaryotes and prokaryotes, including N6-methyladenine (6mA) (Heyn and Esteller, 2015; O’Brown and Greer, 2016), N5-methylcytosine (5mC) (Hu et al., 2013; Syed et al., 2016; Wu and Zhang, 2017), N4-methylcytosine (4mC) (Li et al., 2019), cytosine-N3 Methylation (3mC) (Dukatz et al., 2019), and N1-methyladenine (1mA) (Iyer et al., 2011). Among these types, DNA 6mA modification, which adds a methyl group (CH3) to the sixth position of the purine ring of adenine, was recently reported and investigated in model species of eukaryote, such as *Homo sapiens* (Xiao et al., 2018b), *Mus musculus* (Yao et al., 2017), *Oryza sativa* (Zhou et al., 2018; Zhang Q. et al., 2018), *Arabidopsis thaliana* (Liang et al., 2018), *Caenorhabditis elegans* (Greer et al., 2015), and *Drosophila melanogaster* (Zhang et al., 2015).

6mA and 5mC are two main types of epigenetic marks in eukaryotes. Although the functions of 5mC have been well known, such as transposon suppression, gene regulation, and epigenetic memory maintenance (Jones and Takai, 2001; Jones, 2012; Smith and Meissner, 2013), concrete function investigation of 6mA is rarer because of the low abundance of 6mA and technological limitations on 6mA detection (Liu et al., 2016). Recently, the development of third generation single-molecule sequencing on the PacBio and Nanopore platforms provided advantages for 6mA modification detection at single-nucleotide resolution and single-molecule level (Eid et al., 2009; Xiao et al., 2017; van Dijk et al., 2018). The methylation signal is detected by the variation in interpulse duration between two successive base incorporations during DNA synthesis (Flusberg et al., 2010; Clark et al., 2012; Fang et al., 2012; Schadt et al., 2013). The high-resolution detection of 6mA facilitated the functional resolution of 6mA in model organisms.

DNA 6mA modification mainly plays a role in the regulation of the restriction–modification system in prokaryotes (Luo et al., 2015); Greer et al. (2015) demonstrated that 6mA is present in eukaryotes and integrates environmental stimuli to regulate biological processes in *C. elegans*. Furthermore, 6mA association with gene expression was also reported in *A. thaliana* (Liang et al., 2018), *H. sapiens* (Xiao et al., 2018b), *M. musculus* (Yao et al., 2017), *Chlamydomonas reinhardtii* (Fu et al., 2015), and *O. sativa* (Zhang Q. et al., 2018). For example, 6mA levels are positively correlated with the expression of key stress-related genes in *O. sativa* (Zhang Q. et al., 2018). Clearly, the above studies mainly focused on the profiling and functional analysis of 6mA modification in the nucleus. However, the knowledge about 6mA in cytoplasm is relatively limited, although the mitochondria have a higher 6mA density compared with the nuclear genome (Xiao et al., 2018b). Therefore, whole genomic patterns of 6mA in cytoplasm and its comparison in nucleus need to be further addressed.

The soybean is economically the most important bean in the world and provides vegetable protein for millions of people and ingredients for hundreds of chemical products. Recently, DNA 5mC methylation was found to be associated with seed development and somatic embryogenesis in soybean (An et al., 2017; Ji et al., 2019). However, the studies on DNA 6mA modification in soybean are relatively limited. Thus, we collected the wild (W05) and cultivated (ZhongHuang13, ZH13) soybean sequences (PacBio) and investigated the differences of 6mA modification in cytoplasm and nucleus for each soybean. First, we decoded the whole-genome profiling of 6mA in W05 and ZH13 and then compared 6mA patterns in cytoplasm for each soybean with those in nucleus. Finally, we investigated the relationship between 6mA modification and gene expression using the above two accessions (W05 and ZH13), and its purpose was to uncover the possibly functional differences of 6mA in cytoplasm between W05 and ZH13.

## Materials and Methods

### Data Collection

The raw sequencing reads in h5 format from the PacBio RSII platform (leaf) and the paired RNA-seq datasets (leaf and stem) of W05 (Xie et al., 2019) were downloaded from the NCBI SRA database^1^, whereas the raw sequencing reads in h5 format from the sequel sequencing platform (leaf) and the paired RNA-seq datasets (leaf and stem) of ZH13 (Shen et al., 2018) were downloaded from the Genome Sequence Archive (GSA)^2^. The accession numbers for all the downloaded datasets are listed in Table 1. To investigate the distribution patterns of DNA 6mA modification between nucleus and cytoplasm in soybean, we extracted the nuclear and cytoplasmic chromosomes from the Ensembl plants database^3^ and the GSA database^4^, respectively, and these datasets were merged to produce the reference genome for read alignment and downstream analysis (Table 1).

**TABLE 1:** The genomic datasets of W05 (wild) and ZhongHuang13 (ZH13, cultivated) soybeans used in this study.

**Accession**	**Type**	**Sample ID**	**Tissue**	**Platform**	**Size (G)**	**References**
W05	DNA	SAMN09862384	Leaf	PacBio RSII	174.8	Xie et al., 2019
	RNA	SAMN09900999	Leaf	Illumina	4.5	
	RNA	SAMN09901000	Stem	Illumina	4.5	
ZH13	DNA	SAMC044340	Leaf	PacBio Sequel	80.7	Shen et al., 2018
	RNA	SAMC079143	Leaf	Illumina	6.9	
	RNA	SAMC079142	Stem	Illumina	6.7	

### Detection of 6mA in W05 and ZH13

The PacBio SMRT analysis pipeline (version 2.3.0) and SMRT Link platform (version 6.0.0) were used to identify DNA 6mA sites in W05 and ZH13, respectively. Each bax.h5 format file of the raw data for W05 was first aligned to the merged reference genome using pbalign in base modification identification mode with parameters (–seed = 1, –minAccuracy = 0.75, –minLength = 50, –concordant –algorithmOptions = “-useQuality,” –algorithmOptions = “-minMatch 12 -bestn 10 -minPctIdentity 70.0”). Then, cmph5tools was used to sort post-aligned datasets, and the polymerase kinetics information was loaded after alignment using loadChemistry.py and loadPulses scripts. Finally, we used ipdSummary.py with parameters (–methylFraction, –identify m6A) to detect 6mA sites. For the ZH13 dataset, the modification_detection workflow mode of pbsmrtpipe (version 0.66.0) was used to detect DNA 6mA sites using the default parameters.

### Bioinformatics Analysis

Circos version 0.69 was used to depict 6mA density and methylation fraction across the nuclear and cytoplasmic genomes (Krzywinski et al., 2009). The 4 bp upstream and downstream flanking each 6mA site were used to perform MEME-CHIP with default settings in order to predict the conservative motif of 6mA sites (Ma et al., 2014; Liang et al., 2018; Zhang Q. et al., 2018; Li et al., 2020). According to the annotation of soybean genome, we divided the methylated nuclear gene regions into 5′ untranslated region (UTR), 3′ UTR, exons (exclude UTRs), and introns as described in literatures (Liang et al., 2018; Zhang Q. et al., 2018; Xie et al., 2019). Similarly, the methylated cytoplasmic genes were separated into exons and introns according to the genome annotation in order to investigate 6mA distribution in gene features.

### Relationship Between 6mA Modification and Gene Expression

To explore the relationship between 6mA modification and gene expression, RNA-seq raw reads were aligned to the merged genome using TopHat version 2.1.1 (Kim et al., 2013). The abundance of gene expression was calculated by cufflinks version 2.2.1 (Trapnell et al., 2010) using the fragments kilobase of exon model per million mapped reads (FPKM). We used R version 3.6.1 to perform the statistical analysis and prepare figures.

## Results

### Comparison of DNA 6mA Distribution in W05 and ZH13

**FIGURE 1:**
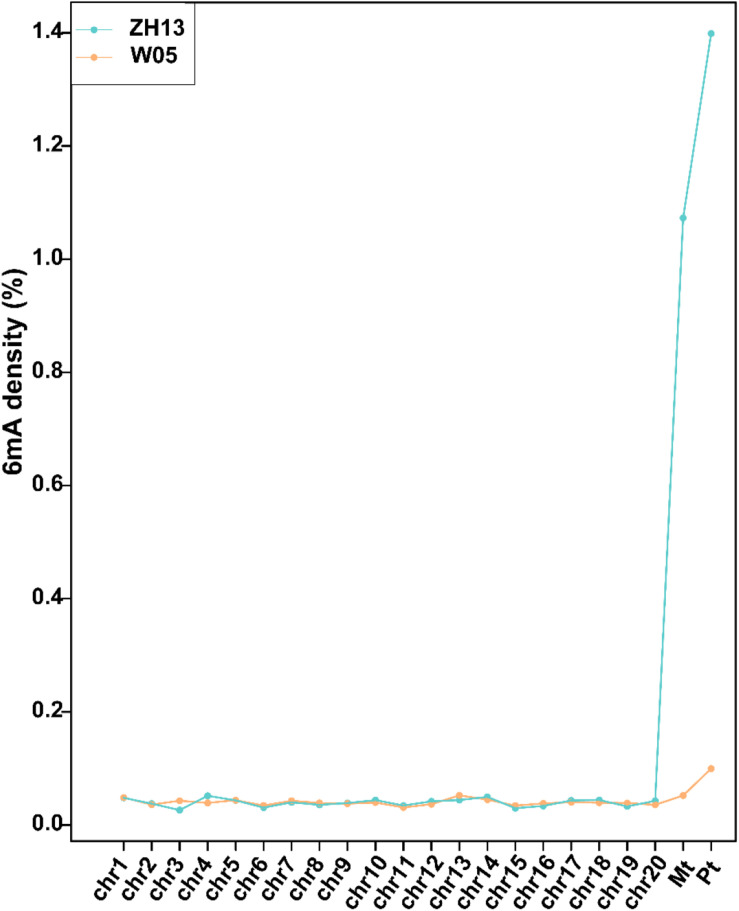
The densities of 6mA on nuclear chromosomes (chr1-20) and mitochondrial (Mt) and chloroplast (Pt) genomes in W05 (wild) and ZH13 (cultivated).

We analyzed the SMRT sequencing data and detected 243,300 and 247,122 DNA 6mA sites in W05 and ZH13, respectively (Supplementary Table S1 and Supplementary Excel S1). The densities of 6mA (6mA/A) for W05 and ZH13 were calculated. As a result, similar genomic 6mA densities for W05 (0.0399%) and ZH13 (0.0406%) were observed (Supplementary Table S1). This result was consistent with that in *A. thaliana* (0.04%) (Liang et al., 2018), but lower than that in *O. sativa* (0.15–0.55%). Meanwhile, 6mA was widely distributed across all 20 autosomal chromosomes in wild and cultivated soybeans, and the 6mA density in autosomal chromosomes ranged from 0.0311% to 0.0524% (W05) and from 0.0264% to 0.0516% (ZH13) (Supplementary Excel S2). Interestingly, DNA 6mA patterns in cytoplasm were different from those in nuclear chromosomes (Supplementary Excel S2), and the mitochondrial and chloroplast genomes in W05 and ZH13 had a higher 6mA density than the autosomal chromosomes (Figure 1 and Supplementary Excel S2). 6mA densities in the autosomal chromosomes were similar across W05 and ZH13, but ZH13 had an extremely high 6mA density in the mitochondrial and chloroplast genomes as compared with W05 (Figure 1). This result indicates that 6mA densities in nuclear genome between wild and cultivated soybeans were similar, but 6mA densities in cytoplasmic genome between the two kinds of soybeans greatly varied. This may be caused by different selection patterns between nuclear and cytoplasmic genomes during domestication (Fang et al., 2016).

**FIGURE 2:**
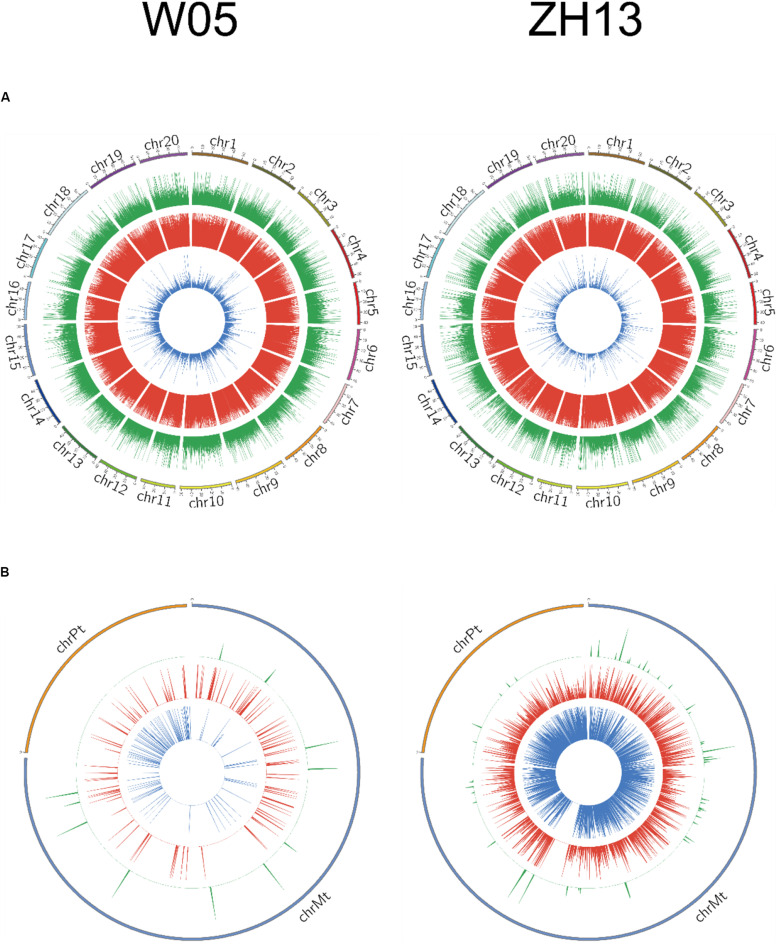
Circos plots of 6mA density distribution in W05 (wild) and ZH13 (cultivated) soybeans in **(A)** nucleus (chromosomes 1–20) and **(B)** cytoplasm (chrPt, chloroplast; chrMt, mitochondria). Blue, red, and green represent low (0–30%), moderate (30–70%), and high (70–100%) methylation fractions, respectively.

The methylation levels were divided into three categories based on the methylation fraction of 6mA sites: low (0–30%), middle (30–70%), and high (70–100%). The concentric rings of circos represented the methylation fraction distribution of 6mA between autosomal and cytoplasmic chromosomes (Figure 2). The middle and high methylated fractions were dominant in the nuclear genomes of W05 and ZH13 (Figure 2A), whereas the low and middle methylated fractions were prevalent in the cytoplasmic genomes of W05 and ZH13 (Figure 2B). In addition, the whole genomic pattern of 6mA density differed between the cytoplasmic and nuclear genomes for each accession (W05 or ZH13) (Supplementary Figure S1). The 6mA density distribution in autosomal genome was enriched at a single region on each chromosome, which was inconsistent with the pattern in the cytoplasmic genome.

### Comparison of 6mA Consensus Motifs in W05 With Those in ZH13

**FIGURE 3:**
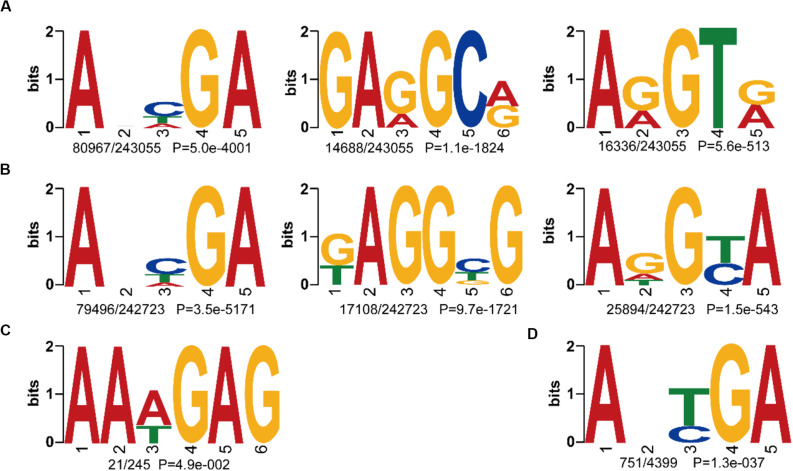
The identified 6mA consensus motifs in W05 (wild) and ZH13 (cultivated) soybeans. **(A)** Nuclear motifs in W05; **(B)** nuclear motifs in ZH13; **(C)** cytoplasmic motifs in W05; **(D)** cytoplasmic motifs in ZH13. The number of times each motif occurred relative to the total number of 6mA-containning motifs and the corresponding *P* value calculated from MEME are shown under the sequence logos.

To further compare the consensus sequences between wild and cultivated soybeans, we extracted the 4 bp upstream and downstream flanking regions of each 6mA site and identified the motif sequences in W05 and ZH13. In the nuclear genomes of W05 and ZH13, the results showed that ANHGA was the most prevalent 6mA motif, accounting for approximately one-third of all identified sites in both W05 and ZH13, whereas GARGCR and ARGTR were significantly enriched in W05, and KAGGBG and ADGYA in ZH13 (Figures 3A,B). To investigate the variance in 6mA motifs between nucleus and cytoplasm, we also analyzed the motifs of 6mA sites in the cytoplasmic genome. As a result, the motif sequence ANYGA in the ZH13 cytoplasm was similar to that in the ZH13 nucleus (Figure 3D), but the motif sequence AAWGAG in the W05 cytoplasm was different from that in the W05 nucleus (Figure 3C). The motif is presumed to have biology function, indicating the critical and conserved roles of the consensus motif in nucleus. Thus, the different cytoplasmic motifs between wild and cultivated soybeans imply different functions of 6mA in the cytoplasm between wild and cultivated soybeans.

### Comparison of 6mA Distribution in Gene Features Between Nucleus and Cytoplasm

**FIGURE 4:**
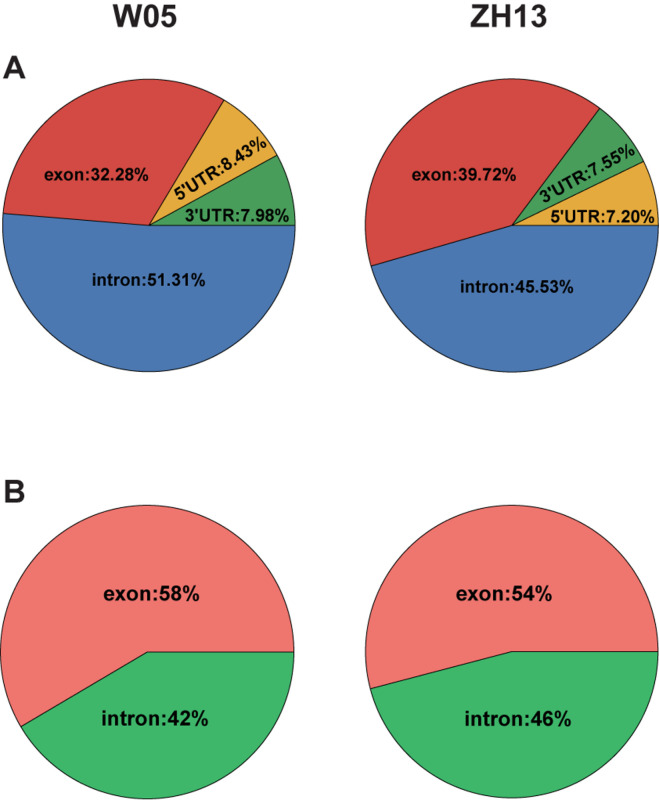
The 6mA distribution in gene features in the nuclear **(A)** and cytoplasmic **(B)** genomes of W05 (wild) and ZH13 (cultivated) soybeans.

To compare the 6mA distribution pattern in the nuclear and cytoplasmic genomes of W05 and ZH13, we analyzed 6mA modification distribution in intergenic regions and gene bodies, as well as their subregions. As a result, 12.76% and 10.73% 6mA sites in the nuclear genomes were located within gene bodies in W05 and ZH13, respectively, whereas the percentage of 6mA sites in the cytoplasmic genomes was 43.27% in W05 and 45.56% in ZH13, being significantly higher than those in the nuclear genomes (*P* = 2.2e-16) (Supplementary Excel S3). We further divided the gene bodies into 5′ and 3′ UTRs, exons (exclude UTRs) and introns, and χ^2^ test was taken to test the difference of 6mA distribution in exon of nucleus and cytoplasm in W05 and ZH13. As a result, significant differences were observed in W05 (*P* = 4.279e-05) and ZH13 (*P* = 1.400e-10). In the nuclear genome, most 6mA sites were enriched in introns (51.31% for W05 and 45.53% for ZH13) (Figure 4A), whereas in the cytoplasmic genome, more than half of the 6mA sites were located in exons (Figure 4B). This indicates that 6mA modification distribution in nuclear genome was different from that in cytoplasmic genome. This enlightens us: 6mA might take effect through different pattern in nucleus and cytoplasm.

We examined the detailed distribution of 6mA sites in nuclear and cytoplasmic genes of W05 and ZH13. As a result, the nuclear and cytoplasmic genes of W05 and ZH13 had the same trend and were enriched in one to three sites (Supplementary Figures S2A,B). Meanwhile, the number of genes with 6mA sites in the nucleus was higher for W05 than for ZH13 (Supplementary Figure S2A), whereas the number of genes with 6mA sites in the cytoplasm was higher for ZH13 than for W05 (Supplementary Figure S2B).

### The Role of 6mA in Gene Expression in W05 and ZH13

**FIGURE 5:**
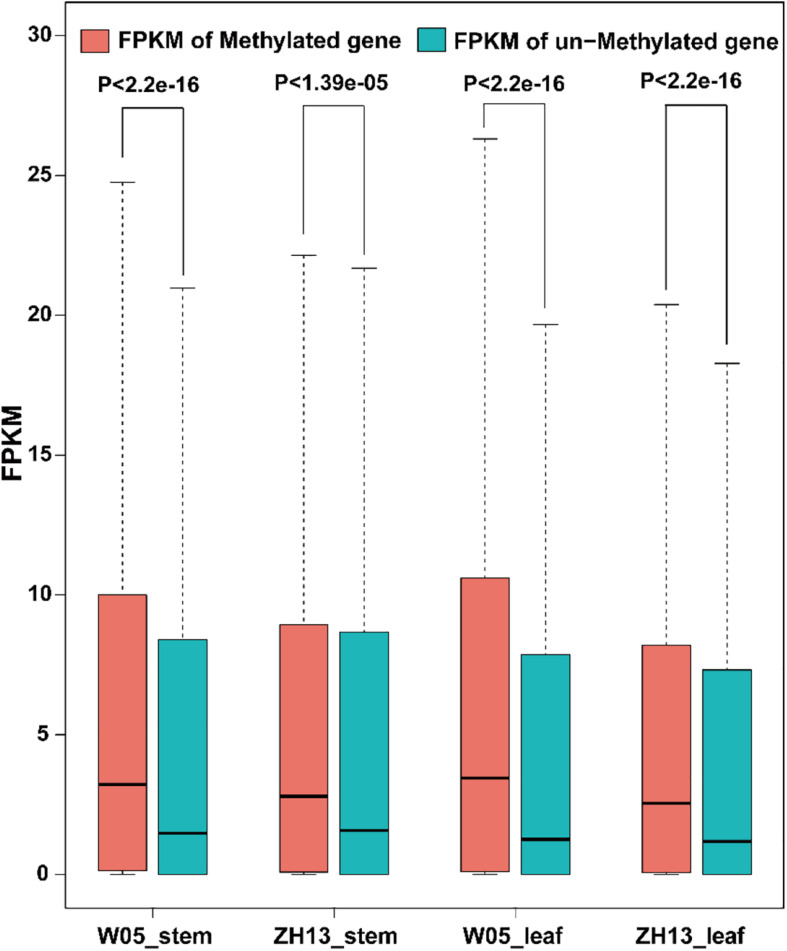
The FPKM values of methylated and unmethylated genes in W05 (wild) and ZH13 (cultivated) soybean genomes.

To examine the relationship between gene expression and 6mA modification, we divided genes into methylated genes and unmethylated genes. For the two kinds of genes, their FPKM values in stem and leaves of W05 and ZH13 were calculated and compared. In the nuclear genome; as a result, methylated genes had a significantly higher expression level in stem and leaves of W05 and ZH13 than unmethylated genes (Figure 5 and Supplementary Excel S4). In cytoplasmic genome, different results were observed. In other words, no significant differences between the expressional levels of methylated and unmethylated genes were observed in the W05 cytoplasm, although methylated genes in the ZH13 cytoplasm had a significantly higher expression level in stem and leaves than unmethylated genes (Supplementary Figure S3). This means that wild and cultivated soybeans have different relationships between 6mA modification and cytoplasmic gene expression.

## Discussion

DNA 6mA modification plays a crucial role in regulating biological processes in eukaryotes (Heyn and Esteller, 2015; Wang et al., 2017; He et al., 2019; Liu et al., 2019; Luan et al., 2019). Genome-wide 6mA distribution has been depicted in plants, such as *A. thaliana* (Liang et al., 2018) and *O. sativa* (Li et al., 2012; Zhang Q. et al., 2018). Soybean is an important crop, however, the whole-genome distribution pattern of 6mA has not yet been investigated. Here, we decoded genome-wide 6mA sites at single-nucleotide resolution with SMRT sequencing data in W05 and ZH13 and found that 6mA sites were extensively distributed across the genome. In order to compare the two kinds of soybeans, W05 (wild) and ZH13 (cultivated), we first reported the discrepancies in 6mA distribution and density between the nuclear and cytoplasmic genomes. The motifs of 6mA in the cytoplasm had a higher variance compared with those in the nucleus. The relationship between 6mA modification and cytoplasmic gene expression differed between wild and cultivated soybeans.

When we compared 6mA density across the chromosomes (Figure 1), we found that the mitochondria and chloroplast had higher 6mA densities than autosomal chromosomes. This is consistent with human 6mA density distribution reported by Xiao et al. (2018b). The DNA deposited in nucleus with double helix pattern, whereas in chloroplast and mitochondria, it was circular. It had been reported that G-quadruplex DNA secondary structures influenced methylation at CpG islands (Mao et al., 2018); a strong dependence of methylation on the topology of CpG had also been studied in human (Lovkvist et al., 2016). Therefore, the different structure of DNA in nucleus and cytoplasm might be one reason associated with the difference of 6mA density between nucleus and cytoplasm. Moreover, 6mA density and distribution in the autosomal chromosomes between wild and cultivated soybeans were similar, but they were different in the chloroplast and mitochondrial genomes (Figures 1, 2). This difference may be associated with the domestication of soybean. Domestication has been reported to alter DNA methylation profiles (Li et al., 2012; Eichten et al., 2013), and chloroplast genes exhibited different selective patterns from those of nuclear genes during soybean domestication (Fang et al., 2016). This may be one reason for the vast discrepancy in 6mA in the cytoplasmic genome, whereas similar features were found in the nuclear genome of wild and cultivated soybeans.

A motif is a short DNA fragment that occurs extensively in the genome and is speculated to have a biological function. Here, we identified ANHGA as the most prevalent motif in the nuclear genome of the two kinds of soybeans. The other top motifs in W05 were GARGCR and ARGTR, whereas it was KAGGBG and ADGYA in ZH13. This indicates that the 6mA motif pattern in the nucleus was predominantly conserved between the two kinds of soybeans, and the limited discrepancies between the two accessions might be caused by the differences between the accessions. ANHGA is similar to ANYGA, which has been identified as the most dominant motif in *A. thaliana* (Liang et al., 2018), and ARGTR overlaps the ARGT motif, which was found in *Xanthomonas oryzae* pv. *Oryzicola* (Xiao et al., 2018a). The above results reveal that the 6mA motif pattern is common among species. Motif analysis in the cytoplasm revealed that AAWGAG was the only cytoplasmic motif in W05, and ANYGA was the most predominant motif in the ZH13 cytoplasm. Motif sequence variance between ZH13 and W05 in the cytoplasm was larger than that in the nuclear genome, indicating that the consistent motif found in the nucleus of W05 and ZH13 might have critical and conserved functions.

Compared with the unmethylated genes, methylated genes had higher expression in the ZH13 cytoplasm, which is consistent with that in humans (Xiao et al., 2018b). In the W05 cytoplasm, however, similar expression levels of methylated and unmethylated genes were observed. These results indicate that 6mA in the cytoplasm might undertake different functions in different accessions, which should be investigated in the future.

## Data Availability Statement

The PacBio and RNA-seq datasets of W05 were downloaded from the NCBI SRA database (https://www.ncbi.nlm.nih.gov/sra/, accession nos: SAMN09862384, SAMN09900999, and SAMN09901000). The PacBio and RNA-seq datasets of ZH13 were downloaded from the GSA database (https://bigd.big.ac.cn/gsa/browse/CRA001007, accession no: SAMC044340; https://bigd.big.ac.cn/gsa/browse/CRA001810, accession nos: SAMC079143 and SAMC079142).

## Author Contributions

S-QX and Y-MZ conceived the project and designed the experiments. D-HY, J-FX, K-KJ, and JG collected the datasets and performed the bioinformatics analysis. D-HY, J-FX, and M-WL plotted the figures. D-HY, S-QX, and Y-MZ wrote the manuscript. All authors read and approved the final manuscript.

## Conflict of Interest

The authors declare that the research was conducted in the absence of any commercial or financial relationships that could be construed as a potential conflict of interest.

## Footnotes

1 https://www.ncbi.nlm.nih.gov/sra/

2 https://bigd.big.ac.cn/gsa/browse/CRA001810

3 http://plants.ensembl.org/Glycine_max/Info/Index

4 https://bigd.big.ac.cn/search?dbId=gwh&q=GWHAAEV00000000.1
