# Supplementary material for: DNA N6-Methyladenine Modification in Wild and Cultivated Soybeans Reveals Different Patterns in Nucleus and Cytoplasm
Source: Front Genet. 2020 Jul 27;11:736. doi: 10.3389/fgene.2020.00736 (PMC7398112; doi:10.3389/fgene.2020.00736)
Supplement: Supplementary file 5 [file Presentation_1.pdf]

Table. S1 Statistical overview of 6mA modification in genome of two varieties.

varieties	Total A number	6mA number	6mA ratio
W05	609,412,990	243,300	0.0399%
ZH13	609,412,990	247,122	0.0406%

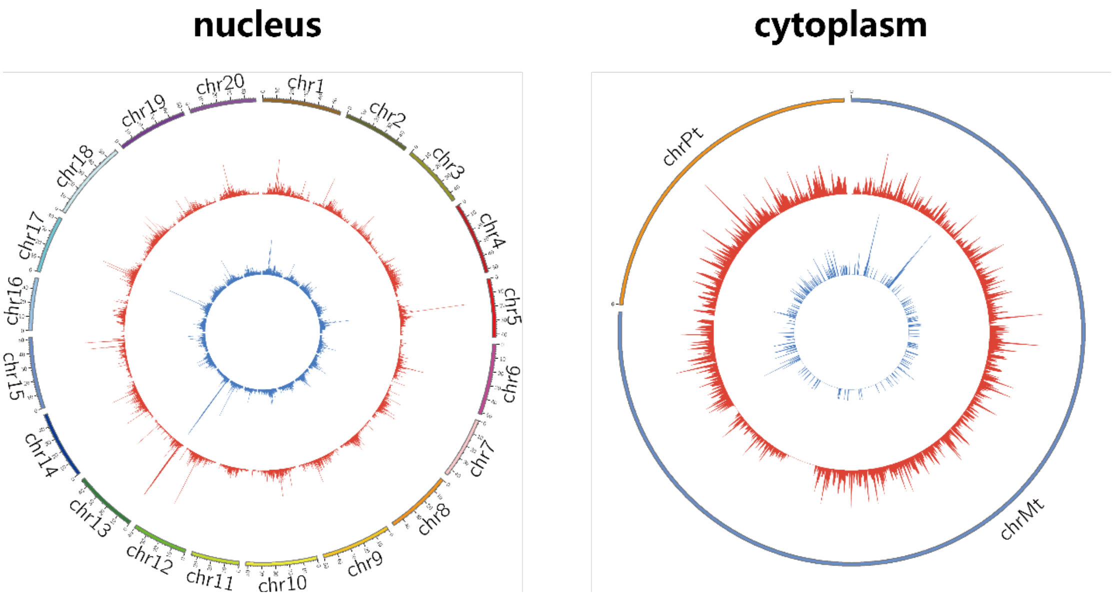

Figure. S1 The 6mA density among soybean genome. Blue, red represent W05 and ZH13, respectively. Left panel is nuclear chromosomes, right panel is cytoplasmic chromosomes.

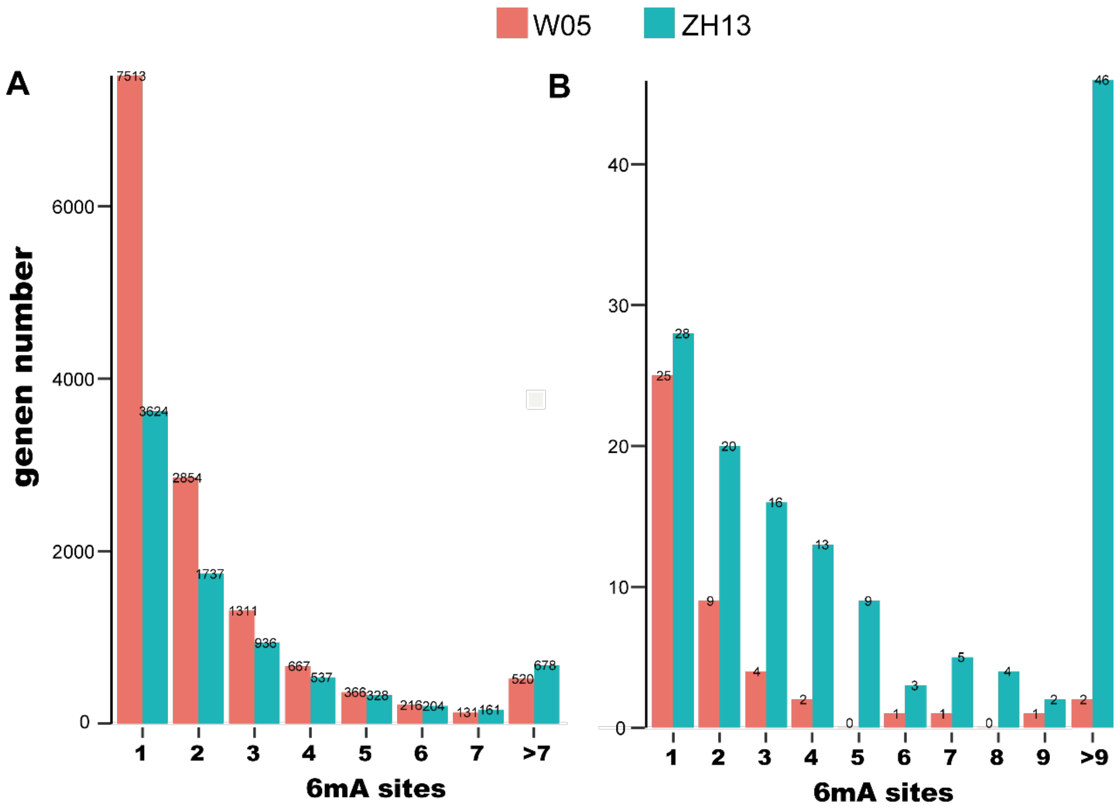

Figure. S2 the gene number with 6mA sites (A: genes in nucleus and B: genes in cytoplasm).

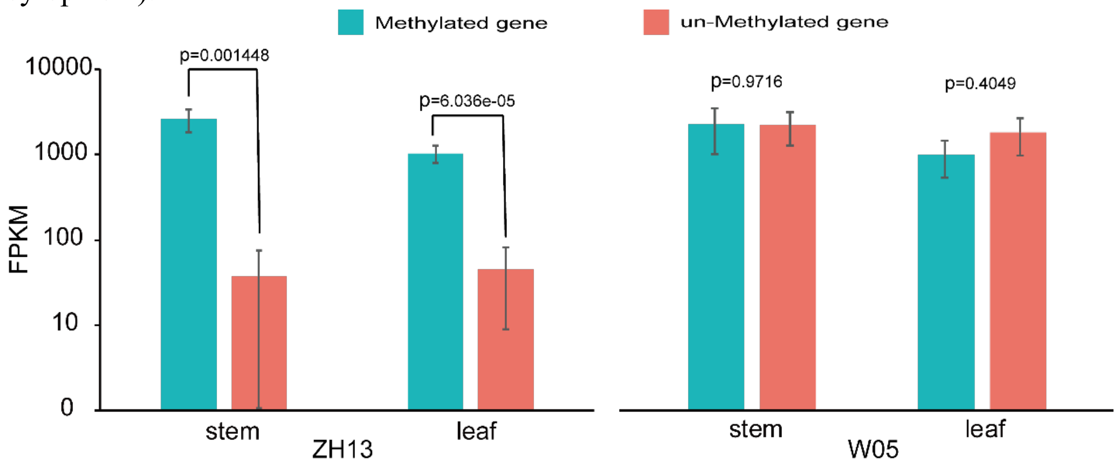

Figure. S3 comparison of the FPKM between methylated gene and un-methylated gene of cytoplasm.
